# Ultrasound-Guided PECS II Block Reduces Periprocedural Pain in Cardiac Device Implantation: A Prospective Controlled Study

**DOI:** 10.3390/medicina61081389

**Published:** 2025-07-30

**Authors:** Mihaela Butiulca, Florin Stoica Buracinschi, Alexandra Lazar

**Affiliations:** 1 Department of Anesthesiology and Intensive Care Medicine, Faculty of General Medicine, George Emil Palade University of Medicine, Pharmacy, Science, and Technology of Targu Mures, 540001 Târgu Mureș, Romania; alexandralazar7@gmail.com; 2 Department of Anesthesiology and Intensive Care Medicine, Emergency County Hospital, 540001 Târgu Mureș, Romania; 3 Department of Medical Informatics and Biostatistics, Faculty of General Medicine, George Emil Palade University of Medicine, Pharmacy, Science, and Technology of Targu Mures, 540001 Târgu Mureș, Romania; florin.stoica@umfst.ro; 4 Department of Internal Medicine, Emergency County Hospital, 540001 Târgu Mureș, Romania

**Keywords:** regional anesthesia, PECS II, CIED

## Abstract

*Background and Objectives*: Implantation of cardiac implantable electronic devices (CIEDs) is increasingly performed in elderly and comorbid patients, for whom minimizing perioperative complications—including pain and systemic drug use—is critical. Traditional local infiltration often provides insufficient analgesia. The ultrasound-guided PECS II block, an interfascial regional technique, offers promising analgesic benefits in thoracic wall procedures but remains underutilized in cardiac electrophysiology. *Materials and Methods:* We conducted a prospective, controlled, non-randomized clinical study including 106 patients undergoing de novo CIED implantation. Patients were assigned to receive either a PECS II block (*n* = 53) or standard lidocaine-based local anesthesia (*n* = 53). Pain intensity was assessed using the numeric rating scale (NRS) intraoperatively and at 1, 6, and 12 h postoperatively. Secondary outcomes included the need for rescue analgesia, procedural duration, length of hospitalization, and patient satisfaction. *Results:* Patients in the PECS II group reported significantly lower NRS scores at all time points (mean intraoperative score: 2.1 ± 1.2 vs. 5.7 ± 1.6, *p* < 0.001; at 1 h: 2.5 ± 1.5 vs. 6.1 ± 1.7, *p* < 0.001). Rescue analgesia (*metamizole sodium*) was required in only four PECS II patients (7.5%) vs. 100% in the control group within 1 h. Hospital stay and procedural time were also modestly reduced in the PECS II group. Patient satisfaction scores were significantly higher in the intervention group. *Conclusions:* The ultrasound-guided PECS II block significantly reduces perioperative pain and the need for additional analgesia during CIED implantation, offering an effective, safe, and opioid-sparing alternative to conventional local infiltration. Its integration into clinical protocols for device implantation may enhance procedural comfort and recovery.

## 1. Introduction

Cardiac implantable electronic devices (CIEDs), such as pacemakers and defibrillators, are increasingly implanted in elderly and frail patients with multiple comorbidities [1]. These patients are particularly vulnerable to complications from systemic anesthesia, making the optimization of perioperative pain management a critical priority [2,3].

Traditional local anesthetic techniques, commonly used for device pocket formation and lead tunneling, often fail to provide adequate analgesia throughout the entire procedure, particularly in deeper tissue layers and during subcutaneous dissection [4].

Recent advancements in ultrasound-guided regional anesthesia have enabled more precise and effective analgesic strategies [4,5]. Among these, the pectoral nerve block type II (PECS II) has shown promising results in breast and thoracic surgeries due to its ability to target the lateral and medial pectoral nerves, as well as the intercostobrachial and long thoracic nerves [6,7]. Although initially developed for breast surgery [8], PECS II block has demonstrated efficacy in other interventions involving the anterior thoracic wall, including vascular port insertions and emerging applications in cardiac device implantation [9,10].

Despite anatomical and physiological plausibility, the use of PECS II blocks during CIED procedures remains underutilized. Pilot studies have suggested that PECS II can provide superior pain control, reduce opioid consumption, and improve patient satisfaction compared to conventional local infiltration [11,12].

Furthermore, regional techniques such as PECS II offer a valuable tool in multimodal analgesic protocols aimed at minimizing systemic drug use and accelerating recovery [12].

This prospective controlled study investigates the efficacy and safety of the PECS II block in patients undergoing de novo CIED implantation. We compared PECS II to standard lidocaine infiltration with a focus on intra- and postoperative pain levels, analgesic consumption, procedural duration, and patient-reported satisfaction. Our goal is to provide clinical evidence supporting the integration of this block into routine electrophysiology practice.

## 2. Materials and Methods

### 2.1. Study Design and Ethical Approval

This was a prospective, comparative observational study, performed as part of standard clinical care conducted at the Emergency Institute for Cardiovascular Diseases and Transplantation in Târgu Mureș, Romania, between November 2021 and January 2024. The study was approved by the institutional ethics committee (approval no. 28212/10 November 2021 and 1515/9 December 2021) and conducted in accordance with the Declaration of Helsinki. All patients provided written informed consent prior to enrollment.

### 2.2. Patient Selection

A total of 106 patients undergoing cardiac implantable electronic device (CIED) implantation were enrolled and divided into two groups: those who received a PECS II block (*n* = 53) and those who received standard local infiltration anesthesia (*n* = 53). This distribution enabled a comparative evaluation of the effectiveness of the two analgesic methods in managing postoperative pain in patients with implantable cardiac devices. The inclusion and exclusion criteria are detailed in Table 1. The sample size of 106 patients was determined based on the total number of eligible patients treated during the study period. No formal power calculation was performed, as this was a pragmatic, exploratory, prospective controlled study.

### 2.3. Pectoral Plane Block II Procedure

**Figure 1:**
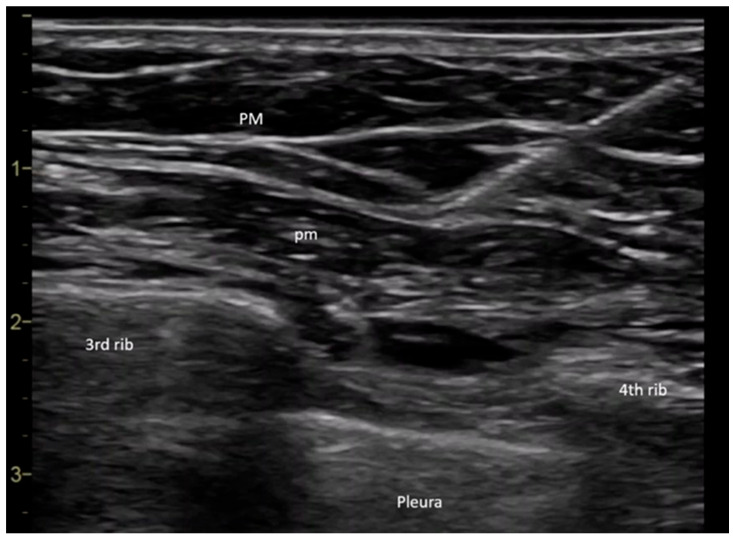
Ultrasonographic image showing the interfascial planes between the pectoralis major and minor muscles during local anesthetic injection (own archive). Legend: PM—pectoralis major muscle, pm—pectoralis minor muscle.

All procedures were performed in the operating room by the same anesthesiologist. Upon the patient’s arrival in the operating room, a peripheral intravenous line was placed, and baseline monitoring was initiated, including pulse, noninvasive blood pressure, and peripheral oxygen saturation measurements. The PECS II block was performed under ultrasound guidance using a General Electric Vivid I6 ultrasound system with an 8 L probe, with the patient positioned in the supine position and the arm placed alongside the body. The ultrasound probe was placed at the level of the coracoid process in the median parasagittal plane. The transducer was then slightly rotated laterally until it reached the deltopectoral groove, where the axillary artery was identified. To ensure the success of the block, it was crucial to locate the ribs. The rib immediately beneath the axillary artery was identified as the second rib. By translating the probe inferiorly and laterally, the third rib appeared in the ultrasound image, allowing for the evaluation of the fascial plane between the pectoral muscles. Before needle insertion through the skin tissue, 2 mL of a local anesthetic mixture was infiltrated using a 27 G needle to provide analgesia at the puncture site. A 20 G plexus needle (Pajunk Sonoplex—Geisingen, Germany 50 mm, 20 G diameter) was then used to puncture the cutaneous plane, with continuous visualization of the needle trajectory. The correct insertion of the plexus needle was confirmed by injecting a saline solution, which produced hydro dissection in the interpectoral plane. Further caudal and lateral translation of the probe allowed for the visualization of the fourth rib. At this point, the serratus anterior muscle could be observed in the ultrasound window. The fascial plane between the pectoralis minor and serratus anterior muscles was the target for the second injection (Figure 1) [13].

An equimolar solution of ropivacaine and lidocaine was infiltrated to ensure the rapid onset of anesthesia. The dose was calculated according to each patient’s ideal body weight. Ropivacaine was administered at a dose of 2 mg/kg, using a solution equimolar to 1% lidocaine. The total dose was calculated based on the patient’s ideal body weight. Sensory block was assessed after 5 min using the cold sensation test.

### 2.4. Implantation Procedure

All implantation procedures were performed by a team of five experienced cardiologists. The implantation technique involved a 5 to 6 cm incision made in the subclavicular region, along the midclavicular line, followed by the dissection of subcutaneous tissue down to the pectoral fascia. Subsequently, tissue dissection and preparation were carried out to create a pocket for the implantable device. The subclavian vein or cephalic vein was then dissected and prepared for lead insertion.

The surgical procedure continued with lead placement, followed by the verification of lead parameters and connection of the leads to the implantable device. The device was inserted into the prepared pocket, and the anatomical layers were sutured. The intervention was completed by suturing the skin and applying sterile dressing.

Throughout the procedure, patients were continuously monitored by an anesthesiologist. Pain assessment was conducted using the Numerical Rating Scale (NRS). After the intervention, patients were transferred to a specialized intensive care unit for post-procedural monitoring. Pain scores were evaluated intraoperatively, immediately post-procedure, and at 1 h, 6 h, and 12 h postoperatively. Rescue analgesia consisted of intravenous administration of metamizole sodium 1 g, given on demand in case of a pain score ≥ 4.

### 2.5. Outcome Measures

The primary outcome was pain intensity, assessed intraoperatively and at 1, 6, and 12 h postoperatively using a numeric rating scale (NRS, 0–10). Secondary outcomes included procedural duration, need for rescue analgesia (Metamizole sodium 1 g), length of hospital stay, and patient satisfaction, evaluated using a postprocedural questionnaire.

### 2.6. Data Collection

The following data were collected from the patients included in the study: demographic and clinical variables such as age, sex, implanted device type, American Society of Anesthesiology (ASA) score, length of hospitalization, and administered anesthetic dose, perioperative pain scores (assessed at multiple time points), comorbidities including pulmonary disease, diabetes mellitus, hypertension, cerebrovascular disease, obesity, and renal disease, and potential complications such as local anesthetic toxicity, pneumothorax, and vascular puncture were monitored.

### 2.7. Statistical Analysis

Statistical analysis was performed using RStudio (version 2024.12.0). Descriptive statistics were used to summarize the data, including continuous, categorical, and binary variables. The Kolmogorov–Smirnov test was applied to assess the normality of continuous variables. Parametric data were expressed as mean ± standard deviation (SD). Non-parametric data were reported as median (interquartile range, IQR). Binary variables were presented as frequencies and percentages. Comparisons of central tendencies were conducted according to the type of variable and its distribution: continuous variables were analyzed using the independent *t*-test for normally distributed data or the Mann–Whitney U test for non-normally distributed data, and binary variables were analyzed using the Chi-square test or Fisher’s exact test, as appropriate. Correlation analysis was performed to identify relationships between variables: Pearson’s correlation was used for parametric variables and Spearman’s correlation was used for non-parametric variables. The correlation coefficients (r), determination coefficient (r^2^), and p-values were reported. A significance level of α = 0.05 was used for all statistical tests.

## 3. Results

### 3.1. Groups Characteristics

We compared the demographic, clinical, and procedural characteristics of the two study groups: the PECS II group (*n* = 53) and the control group (*n* = 53). The data presented in Table 2 highlight the overall similarities between the two groups. The mean age of the patients was comparable: 66.00 ± 12.62 years in the control group and 66.96 ± 11.13 years in the ropivacaine group. The sex distribution was relatively balanced, with 33 males (62.26%) and 20 females (37.73%) in the control group, versus 29 males (54.71%) and 24 females (45.28%) in the ropivacaine group. Notably, the PECS II group included more patients with an ASA IV score (30 patients, 56.60%) compared to the control group (21 patients, 39.62%), suggesting a worse overall health status in this group. Conversely, the control group had a higher proportion of ASA III patients (32 patients, 60.37%) compared to the PECS II group (23 patients, 43.39%). The differences between the two groups were not statistically significant, indicating their homogeneity and allowing for an objective comparison of the results regarding the efficacy of analgesia.

### 3.2. Pain Intensity Analysis

**Figure 2:**
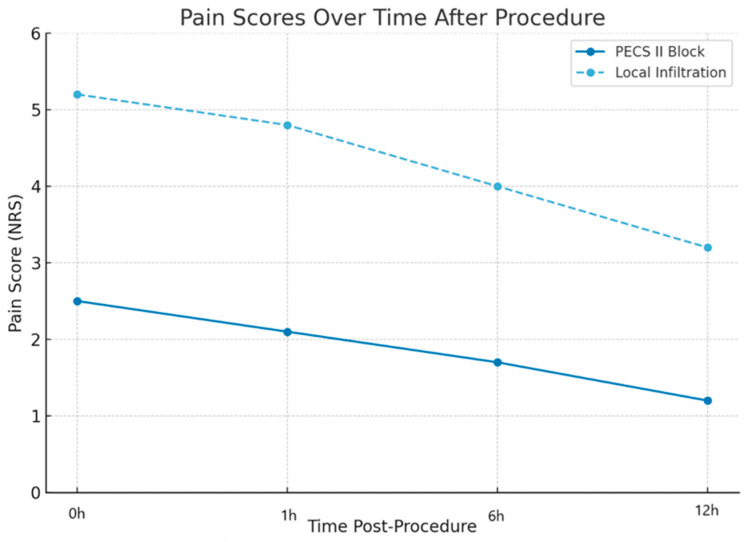
Temporal evolution of pain scores in both groups.

Pain scores were significantly lower in the PECS II group at all measured time points. The mean intraoperative NRS score was 2.1 ± 1.2 in the PECS II group compared to 5.7 ± 1.6 in the control group (*p* < 0.001). At 1 h post-procedure, PECS II patients reported a mean score of 2.5 ± 1.5 versus 6.1 ± 1.7 in the control group (*p* < 0.001). This trend persisted at 6 and 12 h, with mean NRS scores of 2.3 ± 1.4 and 2.0 ± 1.1 in the PECS II group, compared to 5.6 ± 1.8 and 4.9 ± 1.6 in the control group, respectively (*p* < 0.001 for all). Figure 2 illustrates the temporal evolution of pain scores in both groups.

There was no significant difference in hospital stay between the two groups (PECS II: 1.3 ± 0.6 days vs. Control: 1.4 ± 0.7 days; *p* = 0.619). Minimum hospitalization duration: 2 days (control group), 3 days (PECS II group). Maximum hospitalization duration: 19 days (control group), 16 days (ropivacaine group).

The distribution of comorbidities was approximately similar between the two groups. The most frequent comorbidity was hypertension (*n* = 31 in the control group, *n* = 34 in the ropivacaine group). Diabetes mellitus was evenly distributed (*n* = 17 in both groups). The control group had more patients with cerebrovascular disease (*n* = 20), chronic kidney disease (*n* = 12), and pulmonary disease (*n* = 16) than the ropivacaine group (*n* = 19, *n* = 11, and *n* = 13, respectively). Obesity was present in 21 patients from the control group and 22 from the PECS II group.

**Figure 3:**
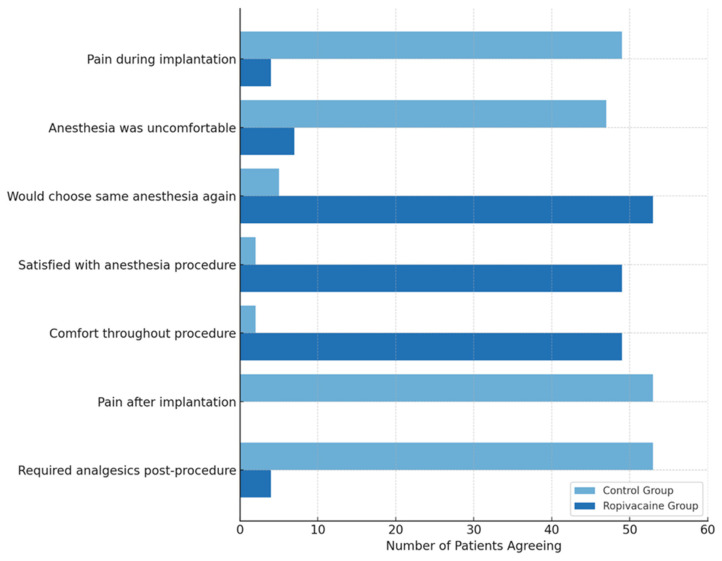
Patient Satisfaction Survey Results.

After the nerve block, all patients exhibited complete sensory block between T2 and T4 dermatomes. In the PECS II group, only 5 patients (9.4%) required additional anesthesia, primarily in cases where the cephalic vein was used for lead implantation, suggesting technical difficulty in these cases. To assess post-procedural satisfaction, patients completed a questionnaire with seven questions. The distribution of patient responses is available in Figure 3. There were statistically significant differences between the two groups, highlighting the superiority of using the pectoral block for procedural anesthesia.

No adverse events related to the regional technique were observed. Specifically, there were no cases of local anesthetic systemic toxicity (LAST), pneumothorax, or inadvertent vascular puncture in either group.

## 4. Discussion

This study aimed to evaluate the efficacy of the PECS II block as an analgesic technique for implantable cardiac device placement, focusing particularly on its ability to effectively control pain without the need for additional anesthetic agents or rescue medication. The results provide preliminary evidence supporting the efficacy of the PECS II block in significantly reducing both intraoperative and postoperative pain, compared to standard lidocaine-based local anesthesia. The study maintained a clinical focus, emphasizing pain management and procedural efficiency.

Patients who received the PECS II block reported lower pain scores both during and after the procedure, compared to the control group. Additionally, the PECS II block was associated with shorter surgical durations and reduced hospitalization times, demonstrating the superior procedural efficiency associated with PECS II blocks, likely due to a reduced need for anesthetic reinfiltration. It has practical benefits for both patients and healthcare systems. These findings suggest that the PECS II block could be integrated as part of a standardized pain management protocol for patients requiring implantable cardiac devices, reducing opioid analgesic dependence and enhancing postoperative recovery.

Compared to previous research, our findings are consistent with those reported by Markman et al. [5], who demonstrated that the ultrasound-guided intraoperative PECS II block resulted in minimal postoperative pain scores of 0.4 ± 0.8 within the first 4 h and 0.3 ± 0.6 at 24 h post-procedure. These findings align with our data, in which patients who received the PECS II block reported significantly lower pain scores and reduced the need for additional analgesics.

Similarly, our results are comparable to those of Kilin et al. [3], who observed a high efficacy of the PECS II block for implantable cardiac devices, although they noted that anesthetic supplementation was necessary in some cases. In contrast to the findings of Mavarez et al. [14], who described an isolated case of successful PECS II block use without supplementation, our study highlights that the block alone is not sufficient in all cases, particularly in patients with complex anatomies or comorbidities.

Another relevant study by Smith et al. [15] emphasized the use of PECS II blocks in cardiothoracic surgery, showing reduced opioid use and faster patient recovery. This supports our findings regarding the benefits of the PECS II block in reducing hospitalization duration and improving intraoperative and postoperative analgesia management.

However, the available literature remains limited, as the exploration of interpectoral blocks for surgical interventions beyond breast surgery has only recently gained interest. Our results contribute to this emerging field, providing additional evidence on the safety and efficacy of this technique for implantable cardiac device placement. Based on these findings, we recommend the inclusion of the PECS II block in clinical guidelines for cardiac device implantation, given its ability to reduce perioperative pain and systemic analgesic requirements. Additionally, training anesthesiologists in ultrasound-guided administration is crucial for ensuring safe and effective implementation.

### Limitations of the Study

This study acknowledges several limitations that should be considered. While adequate for preliminary analysis, sample size limits the generalizability of the results. No direct comparisons were made with general anesthesia or other regional techniques, such as paravertebral blocks, restricting a full understanding of the comparative advantages of the PECS II block. Differences in administration technique and individual pharmacokinetic variability of ropivacaine could influence analgesic efficacy, requiring further studies to optimize procedural parameters and dosing protocols. The study did not include long-term patient monitoring for evaluating chronic pain or other postoperative complications, representing another significant limitation.

Additionally, this study was designed as a non-randomized controlled trial. Although the two groups were comparable in terms of demographic and clinical characteristics, the absence of randomization introduces a potential risk of selection bias. Patient allocation was not blinded, which may have influenced treatment decisions and subjective outcomes, including perceived pain and satisfaction levels. Future randomized controlled trials are necessary to confirm these preliminary findings and eliminate potential biases related to group assignments.

Also, another limitation of this study is the absence of a formal sample size calculation. The study was designed as a pragmatic, prospective trial, and the number of patients included reflects the entire eligible population within the enrollment period. While the sample size allowed the detection of significant differences in primary outcomes, the findings should be interpreted with caution and confirmed in future randomized studies.

In five patients, the cephalic vein was used for venous access, which required a more lateral surgical dissection. These were the only patients in the PECS II group who reported significant intraoperative or postoperative pain, highlighting a potential limitation of the block in covering lateral chest wall innervation. This detail should be considered when planning PECS II for procedures involving extended or lateral dissection.

Future prospective, randomized studies should aim to confirm the observed benefits, investigate long-term outcomes, and refine standardized protocols for optimizing patient outcomes and procedural efficiency. A structured monitoring protocol should be implemented to record operative time in a standardized way, separating block administration, venous access time, and lead placement duration. This would help isolate the true effect of the PECS II block on surgical efficiency and improve comparability across patient cases.

## 5. Conclusions

This study provides evidence supporting the PECS II block as an effective and safe analgesic technique for implantable cardiac placement. Compared to conventional lidocaine-based local anesthesia, this technique demonstrated significant benefits in terms of pain reduction and increased patient comfort, with minimal need for additional analgesic supplementation in most cases. Reduced periprocedural pain and discomfort were reported in patients who received the pectoral block with ropivacaine, compared to the control group. There was a lower requirement for rescue analgesia—only four patients required additional supplementation within the first 12 postoperative hours, compared to all control group patients requiring analgesia within 1 h post-implantation. Shorter procedure duration and hospitalization time were observed, leading to higher patient satisfaction. The PECS II block should be integrated as part of a standardized pain management protocol for patients undergoing implantable cardiac device placement, contributing to reduced opioid dependence and improved postoperative recovery.

## Figures and Tables

**Table 1:** Inclusion and Exclusion Criteria.

Criteria	Description	Justification
Inclusion Criteria		
Age over 18 years	Capacity to sign the informed consent form	Patients must be capable of providing informed consent for study participation
De novo implantation of a CIED	First-time implantation to avoid confounding factors	Ensuring the homogeneity of studied cases
Agreement to participate	Ethical and procedural necessity	Respecting patient rights
Exclusion Criteria		
CIED reimplantation	Patients who had undergone a previous procedure for the same type of device	Avoiding the influence of confounding variables on postoperative pain
Inability to communicate	Patients with severe cognitive or neurological deficits	Ensuring an accurate assessment of perioperative discomfort
Informed refusal	Patients who declined inclusion in the study	Respecting patient rights
Infection at the puncture site	Patients with active infections at the implantation site	Preventing infectious complications

**Table 2:** Baseline Demographic and Clinical Characteristics of Study Groups.

Variable	Control Group (*n* = 53)	PECS II Group (*n* = 53)	*p*-Value
Age (years)	66.00 ± 12.62	66.96 ± 11.13	0.678
BMI (kg/m^2^)	26.9 ± 4.3	27.1 ± 4.1	0.510
Sex (M/F)	33/20	29/24	0.749
Procedure Duration (min)	17.2 ± 3.0	16.8 ± 3.1	0.456
Hospital Stay (days)	1.4 ± 0.7	1.3 ± 0.6	0.619
Hypertension	31	34	0.720
Diabetes Mellitus	17	17	1.000
Renal Disease	12	11	0.788
Obesity	21	22	1.000
Cerebrovascular disease	20	19	1.000
Pulmonary disease	16	13	0.663

## Data Availability

The data presented in this study are available on request from the corresponding author. The data are not publicly available due to privacy or ethical restrictions.

## Abbreviations

The following abbreviations are used in this manuscript:

CIEDs	Implantation of cardiac implantable electronic devices
NRS	Numeric Rating Scale
PECS	Pectoral Plane Block
SD	Standard Deviation
